# Functional dissection of the *PEROXIN11* gene family in *Arabidopsis*

**DOI:** 10.1016/j.abiote.2026.100062

**Published:** 2026-06-17

**Authors:** Wanxin Zhu, Mahmudul Hasan Rifat, Xiaowen Wang, Yihong Fu, Yifang Zhu, Yukang Wang, Li Tai, Shuyan Song, Yanlei Feng

**Affiliations:** a College of Agriculture and Biotechnology, Zhejiang University, Hangzhou, 310058, China; b ZJU-Hangzhou Global Scientific and Technological Innovation Center, Zhejiang University, Hangzhou, 310027, China

**Keywords:** PEX11, Peroxisome proliferation, Functional redundancy, Fatty acid β-oxidation, Photorespiration

## Abstract

Peroxisomes are metabolic organelles in eukaryotes, whose proliferation is regulated by the PEROXIN 11 (PEX11) protein family. Arabidopsis (*Arabidopsis thaliana*) contains five *PEX11* genes that show phylogenetic divergence and distinct expression patterns, but their individual functions remain unclear. Here, we generated a series of higher-order null mutants of the Arabidopsis *PEX11* genes. The combined mutation of all *PEX11* members within each lineage caused no obvious growth or metabolic defects, pointing to functional redundancy throughout the entire family. However, the *pex11abcd* quadruple mutant showed impaired fatty acid β-oxidation and photorespiration, along with significantly reduced peroxisome abundance. The *pex11abcde* quintuple mutant failed to germinate unless its seed coats were manually removed and showed seedling mortality after germination. The results demonstrate that the functions of certain PEX11 proteins in Arabidopsis depend primarily on gene dosage rather than paralog specificity, providing insight into the roles of specific members of this family in maintaining peroxisome abundance and plant viability.

*Dear Editor*,

Peroxisomes function as metabolic hubs in eukaryotic organisms, serving as sites of essential processes such as fatty acid β-oxidation, photorespiration, and phytohormone biosynthesis in plants [1,2]. To sustain metabolic efficiency, peroxisome abundance and proliferation must be tightly regulated [3]. The PEROXIN 11 (PEX11) protein family plays crucial roles in peroxisome membrane elongation and organelle division. These conserved membrane-associated proteins are encoded by genes whose copy number varies across species. Among the three human PEX11 paralogs, PEX11γ is required for peroxisome membrane elongation, while PEX11α and PEX11β mediate peroxisome proliferation under inducing and non-inducing growth conditions, respectively [4]. By contrast, yeast possesses only one PEX11 protein, which acts as a regulatory node that connects membrane remodeling to the activation of the peroxisome division machinery; for example, this protein serves as a GTPase-activating protein for Dynamin-related protein 1 [5,6].

In plants, the PEX11 family participates in peroxisome proliferation in conjunction with several proteins associated with peroxisome fission, including dynamin-related protein 3A (DRP3A), DRP3B, DRP5B, FISSION 1A (FIS1A), and FIS1B [7,8]. Plants generally possess more *PEX11* paralogs than other eukaryotic species; for instance, Arabidopsis (*Arabidopsis thaliana*) possesses five PEX11 family members, PEX11A-E. Individual Arabidopsis *PEX1*1 RNA interference (RNAi) lines showed no discernible phenotypes related to β-oxidation or growth, pointing to functional redundancy among these *PEX11* genes [8]. Overexpression or RNAi of individual *PEX11* genes resulted in various changes in peroxisome morphology and/or abundance [8,9]. In addition, there is evidence for functional specialization among PEX11 paralogs. For example, *At*PEX11B promotes light-induced peroxisome proliferation through FHA3-dependent transcriptional regulation [10], while *At*PEX11E directly interacts with the ENDOSOMAL SORTING COMPLEX REQUIRED FOR TRANSPORT (ESCRT) component FYVE DOMAIN PROTEIN REQUIRED FOR ENDOSOMAL SORTING 1 (FREE1) to facilitate trafficking of the lipase SUGAR DEPENDENT 1 (SDP1) to lipid droplets for lipid degradation [11]. However, we currently lack a comprehensive view of the functions of PEX11 family members in plants.

To date, most functional studies of PEX11 proteins in plants have relied on RNAi, which often leads to only partial suppression of gene expression. To overcome this limitation and to systematically decipher the functions of individual PEX11 family members, we employed an integrated strategy combining phylogenetic, gene expression, and mutant analyses. We performed phylogenetic analysis of PEX11 family proteins using orthologous sequences from diverse eukaryotic lineages. Consistent with a previous report [8], plant *PEX11* genes diverged into three distinct branches following the emergence of land plants. *At*PEX11C, D, and E belong to the same clade, while *At*PEX11A and B belong to two other clades (Fig. S1A). The divergence of PEX11C, D, and E appears to have resulted from a relatively recent duplication event (Fig. S1A). The *At*PEX11 proteins share high sequence similarity (Fig. S1B).

We also analyzed the expression patterns of *AtPEX11* genes throughout development, revealing distinct expression profiles among family members (Fig. S1C). For example, *PEX11A* and *PEX11E* are highly expressed during germination, whereas *PEX11C* is highly expressed in leaves (Fig. S1C). These gene expression patterns suggest that PEX11A and E play more important roles in seed germination through fatty acid β-oxidation, while PEX11C might be crucial for photorespiration in green tissue.

To dissect the functional diversification among PEX11 family members, we used CRISPR-Cas9 and hybridization to generate combinatorial *pex11* knockout mutants (Fig. S2A–B). We obtained mutants from the Arabidopsis Biological Resource Center (ABRC) and screened all mutants by RT-PCR or RT-qPCR to confirm that they were null mutants lacking detectable transcripts of the respective genes (Fig. S2C). The *pex11ab* double and *pex11cde* triple mutants represent mutants of each of the two major evolutionarily divergent clades (Fig. S1A), while the *pex11ae* double and *pex11bcd* triple mutants represent mutants of *PEX11* genes expressed in seeds and leaves, respectively (Fig. S1C). The phenotypes of all these mutants were indistinguishable from those of the wild type (WT) under normal growth conditions. We characterized the physiological consequences of these mutations using assays probing core peroxisomal functions: fatty acid β-oxidation and photorespiration.

Given that the peroxisome serves as the exclusive site for fatty acid β-oxidation and contributes to the catabolism of indole-3-butyric acid (IBA) to indole-3-acetic acid (IAA) in plants, we first examined whether any mutant displayed sucrose-dependent germination, a hallmark of β-oxidation deficiency [1]. Because mutants deficient in fatty acid β-oxidation lack the energy or metabolites needed for proper seedling establishment into a photosynthetic plant, these mutants produce short hypocotyls when grown in the dark, a phenotype that can be rescued by sucrose [1,3]. However, none of the mutants showed differential growth on sucrose-containing versus sucrose-free medium (Fig. 1A–B and S2D). We then assessed peroxisomal β-oxidation efficiency using IBA and 2,4-dichlorophenoxybutyric acid (2,4-DB, a synthetic analog of IBA), both of which require peroxisomal β-oxidation for their conversion into active auxin. Since auxin greatly influences root morphology by inhibiting root elongation, mutants deficient in auxin responses often exhibit long primary roots [12]. Mutants with impaired β-oxidation, such as mutants of the peroxisome protein import factor PEX14, typically exhibit resistance to these compounds due to reduced auxin conversion [13]. Consistent with the results of the sugar-dependence assay, all mutants retained WT sensitivity to both IBA and 2,4-DB (Fig. 1C–D and S2D). These observations suggest that functional redundancy exists across different PEX11 clades, allowing normal peroxisomal function and plant development to be maintained in the mutants.

To fully abolish PEX11 function, we generated a *pex11abcde* quintuple mutant through CRISPR-Cas9 in the *pex11c* mutant background. The germination rate of homozygous mutant seeds was zero (Fig. 1E). Among the progeny from plants heterozygous for the *pex11e* allele in the homozygous *pex11abcd* quadruple mutant background, approximately 25.9% (28/108) of seeds failed to germinate (Fig. S2E), demonstrating that the PEX11 family is essential for Arabidopsis seed germination. The defective germination in several peroxisomal mutants, such as the peroxisomal glyoxylate cycle mutants *cts* and *csy2 csy3*, can be rescued by removing the seed coat [14,15]. Therefore, we manually removed the seed coats from non-germinating *pex11abcde* quintuple mutant homozygous seeds. On medium containing 3% sucrose rather than 1% sucrose, the mutant embryos developed into seedlings (Fig. 1E and S2F). However, these rescued plants exhibited severe developmental defects, including extreme dwarfism and the failure to produce inflorescences (Fig. 1F). Together, these results demonstrate that PEX11 proteins play essential roles in plant development, from seed germination to reproductive growth.

**Fig. 1:**
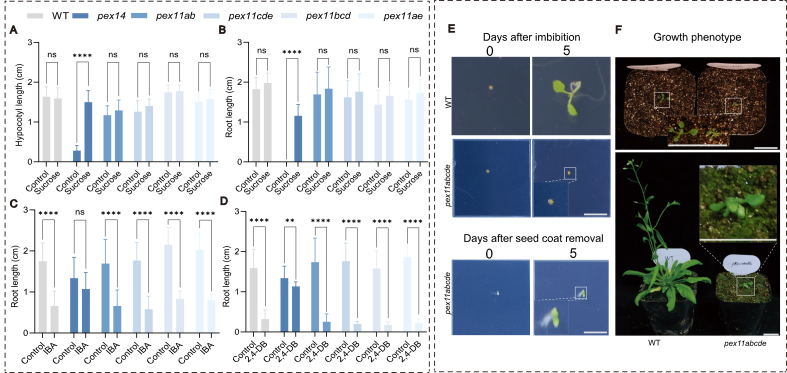
**The PEX11 family is essential for peroxisome function and seedling development in *Arabidopsis*. A-B** Sucrose-dependent growth of *pex11* mutants. Hypocotyl **(A)** and root **(B)** lengths were measured in seedlings grown in the dark or light, respectively, on medium with or without 1% sucrose. **C-D** Peroxisomal β-oxidation assays. Primary root elongation was assessed on medium containing 16 μM IBA **(C)** or 0.8 μM 2,4-DB **(D)**. (A–D) Data are presented as mean ± SD (*n* = 20). Significant differences were determined by two-tailed Student's *t*-test (∗, *P* < 0.05; ∗∗, *P* < 0.01; ∗∗∗, *P* < 0.001; ∗∗∗∗, *P* < 0.0001). **E** Representative images of the WT and *pex11abcde* quintuple mutant. Top panels: germination on 1% sucrose; bottom panels: a rescued quintuple mutant seedling following seed coat removal, grown on 3% sucrose. Bars = 5 mm. The inset shows a 2X enlargement of the image. **F** Phenotypes of the rescued *pex11abcde quintuple* mutant after 2 weeks (top) and 6 weeks (bottom) of growth. The inset shows a 2X enlargement (top) and 5X enlargement (bottom) of the image. Bar = 1 cm.

To further dissect PEX11 function and the seedling mortality of the homozygous *pex11abcde* quintuple mutant, we constructed a homozygous *pex11abcd* quadruple mutant. When *pex11abcd* quadruple mutant seedlings germinated on sucrose-free medium, they displayed significantly shorter hypocotyls and roots than the WT when grown in both the light and dark (Fig. 2A–B). Exogenous sucrose largely rescued this growth defect, confirming that the quadruple mutant failed to efficiently mobilize seed storage lipids to fuel post-germinative seedling development. Similarly, the *pex11abcd* quadruple mutant showed resistance to IBA and 2,4-DB, as reflected by the attenuated inhibition of primary root elongation (Fig. 2C–F). These data demonstrate that the *pex11abcd* quadruple mutant was strongly deficient in fatty acid β-oxidation.

**Fig. 2:**
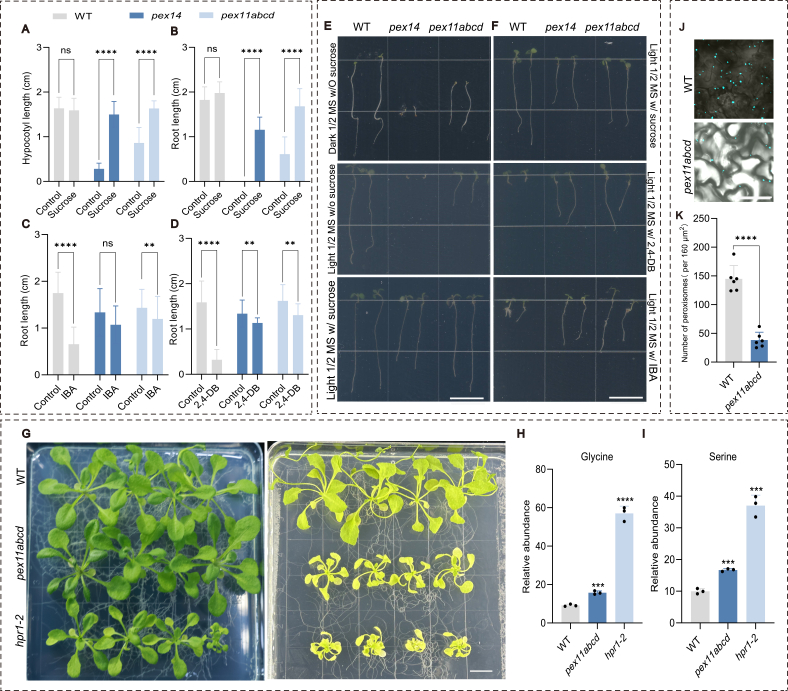
**The quadruple *pex11* mutant exhibits defects in both β-oxidation and photorespiration**. **A** Sucrose dependence assay. Hypocotyl lengths of seedlings grown for 5 day (d) in the dark on half-strength Murashige and Skoog (MS) medium with or without 1% (w/v) sucrose. **B** Same as (A), but root lengths were measured in seedlings grown for 7 d in the light. **C** Effects of IBA on primary root elongation. Plants were grown for 7 d in the light on half-strength MS medium with 16 μM IBA. **D** Effect of 2,4-DB on primary root elongation. Same as (C), but plants were grown on half-strength MS medium with 0.8 μM 2,4-DB. In (A) to (D), all values represent mean ± SD, *n* = 20, and significant differences were determined by two-tailed Student's *t* tests (∗, *P* < 0.05; ∗∗, *P* < 0.01; ∗∗∗, *P* < 0.001; ∗∗∗∗, *P* < 0.0001). **E** Seedlings grown on half-strength MS medium without 1% (w/v) sucrose for 5 d in the dark (top panel) or 7 d in the light (middle panel), and seedlings grown on half-strength MS medium with 1% (w/v) sucrose (bottom panel). Bars = 1 cm. **F** Seedlings grown for 7 d in the light on half-strength MS medium with 0.5% (w/v) sucrose (top panel), half-strength MS medium with 0.5% (w/v) sucrose and 16 μM IBA (middle panel), or half-strength MS medium with 0.5% (w/v) sucrose and 0.8 μM 2,4-DB. Bars = 1 cm. **G** Effects of limited gas exchange conditions on seedling growth. Left panel, plants were grown 27 d on half-strength MS medium with 1% (w/v) sucrose. Right panel, plants were grown under the same conditions, but the plate was sealed after 7 d of growth in a normal environment. Bar = 1 cm. **H–I** Relative abundance of glycine and serine, respectively. All values represent mean ± SD, *n* = 3, and significant differences were determined by two-tailed Student's *t* tests (∗∗∗, *P* < 0.001; ∗∗∗∗, *P* < 0.0001). **J** Representative confocal images of 14-d-old seedlings expressing moxCerulean3-PTS1 in the WT and *pex11abcd* quadruple mutant backgrounds. Bar = 30 μm. **K** Statistical analysis of the number of peroxisomes. All values represent mean ± SD, *n* = 6, and significant differences were determined by two-tailed Student's *t* tests (∗∗∗∗, *P* < 0.0001).

Photorespiration is a major peroxisomal function in photosynthetic tissue that salvages the toxic glycolate produced by the oxygenase activity of the photosynthetic enzyme Rubisco [16,17]. Therefore, we examined photorespiration phenotypes in the *pex11abcd* quadruple mutant. The *pex11abcd* quadruple mutant did not show obvious growth differences from the WT when grown under standard conditions. However, under limited gas exchange conditions, in which the access of CO_2_ to the plants was reduced, this mutant showed pronounced growth retardation, with a phenotype intermediate between that of the WT and the photorespiration-defective *hydroxypyruvate reductase 1* (*hpr1-2*) mutant (Fig. 2G). The levels of the photorespiratory metabolites glycine and serine were also higher in the quadruple mutant compared to WT when grown under limited gas exchange conditions (Fig. 2H–I).

Since PEX11 proteins promote peroxisome proliferation, we reasoned that the pleiotropic defects in the quadruple mutant might stem from reduced numbers of peroxisomes. To test this hypothesis, we introduced a fluorescent peroxisomal marker (moxCerulean3-PTS1) into WT and *pex11abcd* quadruple mutant plants and quantified peroxisome abundance by confocal microscopy. The peroxisome number was significantly reduced in the quadruple mutant compared to WT (Fig. 2J–K), suggesting that the metabolic deficiencies in the mutant were likely due to the lack of a sufficient number of peroxisomes.

Our systematic analysis of the Arabidopsis *pex11* mutant series revealed significant functional redundancy among PEX11 family members. Despite the distinct evolutionary origins and expression patterns of the *PEX11* genes, mutants lacking the functions of individual phylogenetic clades of PEX11 or specific expression domains remained phenotypically indistinguishable from the WT. Significant defects in peroxisome-dependent processes emerged only upon the simultaneous loss of at least four of the five *PEX11* genes, as manifested by impaired fatty acid β-oxidation and compromised photorespiration and plant growth. These metabolic and growth defects were directly associated with a drastic reduction in peroxisome abundance, establishing a clear relationship between *PEX11* gene dosage and the peroxisome population. The failed germination and early seedling mortality of the quintuple *pex11* mutant further underscore the essential nature of PEX11 proteins in plant viability. Together, our findings establish a quantitative, gene-dosage-dependent model for the maintenance of peroxisome function in plants.

These results are broadly consistent with, and extend, recent findings. A newly published study indicates that different Arabidopsis PEX11 paralogs promote the formation of peroxisomal intralumenal vesicles and limit peroxisome size, yet they exhibit functional differentiation in terms of temporal activity [18]. Similarly, complete loss of the PEX11 family leads to seedling mortality. Notably, the *pex11cde* triple mutants described by Tharp et al. [18] displayed pronounced growth defects, whereas their *pex11abcd* quadruple mutant showed only mild phenotypes, a finding that differs slightly from the results of the current study. This difference might stem from the distinct allelic variants employed, which might exhibit gene disruption to different extents. Nevertheless, much remains to be explored regarding the specific functions of Arabidopsis PEX11 proteins. Although functional redundancy is often assumed, their precise contributions to peroxisome fission and other aspects of organelle dynamics require further investigation, particularly given the structural complexity and high plasticity of peroxisomes.

## 1 Materials and methods

### 1.1 Plant materials and growth conditions

All *Arabidopsis thaliana* lines used in this study are in the Col-0 background. The T-DNA insertion mutants *pex11a-1* (SALK_038574) [19], *pex11c-1* (SALK_057358C), *pex11e-1* (SALK_061015), *hpr1-2* (CS799961), and *pex14* (SALK_007441) were obtained from the Arabidopsis Biological Resource Center (https://abrc.osu.edu/). Seeds were surface-sterilized and sown on half-strength MS medium. After stratification at 4 °C in the dark for 3 days, the plates were transferred to growth chambers set to a 16 h/8 h light/dark cycle and a temperature cycle of 22 °C (day) and 20 °C (night).

### 1.2 CRISPR-Cas9 vector construction

A multiplex CRISPR-Cas9 system was used to generate *pex11* mutants. The pYLCRISPR/Cas9Pubi-H vector was employed to express four gRNAs targeting *PEX11A*, *PEX11B*, *PEX11D*, and *PEX11E* simultaneously as previously described [20]. gRNA sequences are listed in Table S1.

### 1.3 Sucrose dependence and IBA/2,4-DB response assays

For sucrose dependence assays, seeds were germinated and grown vertically on half-strength MS medium with or without 1% (w/v) sucrose for 7 days in the light or 5 days in the dark. For IBA and 2,4-DB sensitivity assays, plants were grown vertically on half-strength MS medium supplemented with 0.5% (w/v) sucrose and either 16 μM IBA or 0.8 μM 2,4-DB. Root length (light-grown plants) and hypocotyl length (dark-grown plants) were measured using ImageJ (http://rsb.info.nih.gov/ij/). All seeds were stratified at 4 °C for 3 days before being transferred to the growth chamber.

### 1.4 Reverse transcription-quantitative PCR (RT-qPCR) and RT-PCR

Total RNA was extracted from frozen plant tissues using an RNA Rapid Extraction Kit (Zhejiang Easy-Do Biotechnology Co., Ltd.). For RT-qPCR, 1 μg total RNA was reverse transcribed into cDNA using HiScript III All-in-one RT SuperMix Perfect for qPCR (Vazyme, Nanjing, China). Quantitative PCR was performed using AceQ qPCR SYBR Green Master Mix (Vazyme) on three biological replicates. For semi-quantitative RT-PCR, 0.5 μg total RNA was reverse transcribed, and gene-specific primers (Table S2) were used for amplification.

### 1.5 Sequence alignment and phylogenetic analysis

Amino acid sequence information from plants, fungi, and animals was downloaded from TAIR (https://www.arabidopsis.org/), Phytozome (https://phytozome-next.jgi.doe.gov/), and Ensembl (https://www.ensembl.org/). Homologous PEX11 sequences were retrieved via local BLAST v2.12.0 with an e-value cutoff of 1e-5 [21], using Arabidopsis PEX11 sequences as references. Multiple sequence alignment was performed with MAFFT v7.490 in auto mode [22], and poorly aligned regions were removed using TrimAl v1.4 in gappyout mode [23]. A maximum-likelihood phylogenetic tree was constructed with IQ-TREE v2.0.7 [24] under the LG + F + G4 model and 1000 ultrafast bootstrap replicates. The Arabidopsis PEX11 amino acid sequence alignment was visualized using Jalview v2 [25].

### 1.6 Analysis of photorespiratory phenotypes

Plants were grown under limited gas exchange conditions as previously described [26]. Specifically, to promote photorespiration, after 14 d of growth, the Micropore surgical tape (3 M) that sealed the plates was replaced by two layers of Parafilm M (Bemis) to restrict gas exchange. The plates were incubated in a growth chamber under a 16 h/8 h light/dark cycle with a temperature cycle of 22 °C (day) and 20 °C (night).

### 1.7 Quantification of peroxisomes

Peroxisomes were labeled using a previously established moxCerulean3 peroxisomal marker [27]. The marker was introduced into Arabidopsis plants via *Agrobacterium tumefaciens*-mediated transformation using the floral dip method [28]. Confocal imaging was performed under an Olympus Fluoview FV3000 microscope, using a 445 nm laser for excitation and a 460–500 nm emission window. Peroxisome numbers were quantified using ImageJ (https://imagej.nih.gov/ij/).

### 1.8 Metabolite extraction and quantification

Rosette tissues (∼50 mg) from two or three individual plants were frozen in liquid nitrogen and ground to a fine powder. Glycine and serine were extracted from the samples by adding 500 μL of water, incubating at 95 °C for 10 min with intermittent shaking, vortexing, and centrifugation at 13,800 × *g* for 5 min. The supernatant was collected for metabolite quantification via High-Performance Liquid Chromatography (HPLC). Metabolite levels were calculated relative to an internal standard and normalized to fresh tissue weight.

## CRediT authorship contribution statement

**Wanxin Zhu:** Writing – original draft, Software, Methodology, Data curation. **Mahmudul Hasan Rifat:** Visualization, Software, Methodology. **Xiaowen Wang:** Resources, Formal analysis, Data curation. **Yihong Fu:** Methodology, Investigation. **Yifang Zhu:** Validation, Methodology, Funding acquisition. **Yukang Wang:** Validation, Methodology, Funding acquisition. **Li Tai:** Validation, Methodology, Funding acquisition. **Shuyan Song:** Validation, Resources, Funding acquisition. **Yanlei Feng:** Writing – review & editing, Validation, Conceptualization.

## Declaration of competing interest

On behalf of all authors, the corresponding author states that there is no conflict of interest.

## Data Availability

All data generated during this study are included in the manuscript and Supplementary files.
