# Supplementary material for: Functional dissection of the PEROXIN11 gene family in Arabidopsis
Source: aBIOTECH. 2026 Jun 17;7(3):100062. doi: 10.1016/j.abiote.2026.100062 (PMC13351133; doi:10.1016/j.abiote.2026.100062)
Supplement: Multimedia component 1 [file mmc1.docx]

**Supplementary Figures**


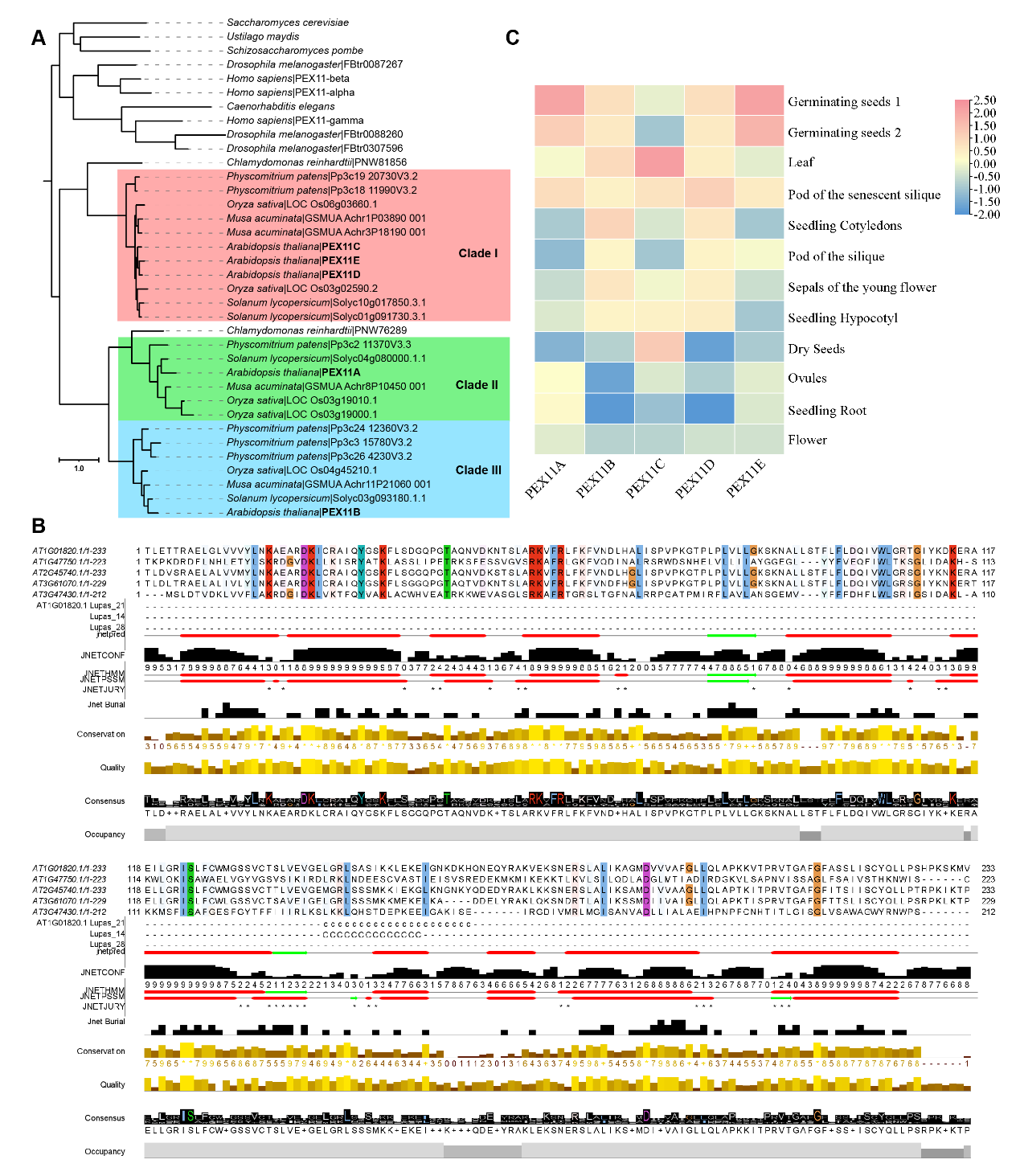


**Fig. S1. Phylogenetic, gene expression, and multiple sequence alignment analyses of PEX11 proteins.**

**(A)** Phylogenetic analysis of the PEX11 family. The maximum-likelihood (ML) tree shows that land plant PEX11 proteins form three distinct clades. For mutant analysis, clades II and III were considered together as one combined lineage, based on which the *pex11ab* mutant was generated. **(B)** Multiple sequence alignment of Arabidopsis PEX11 proteins. Amino acid conservation is color-coded, with secondary structure elements (β-sheets in green, α-helices in red, and coils as black lines) predicted by Jnet. Conservation scores are shown as a gradient from light to dark yellow. **(C)** Expression of *PEX11* genes across various developmental stages of Arabidopsis. The expression is presented in log2 scale. The clade of the left side presents the relation between the different developmental stage expression level, all data are from TAIR (https://www.arabidopsis.org/).

**
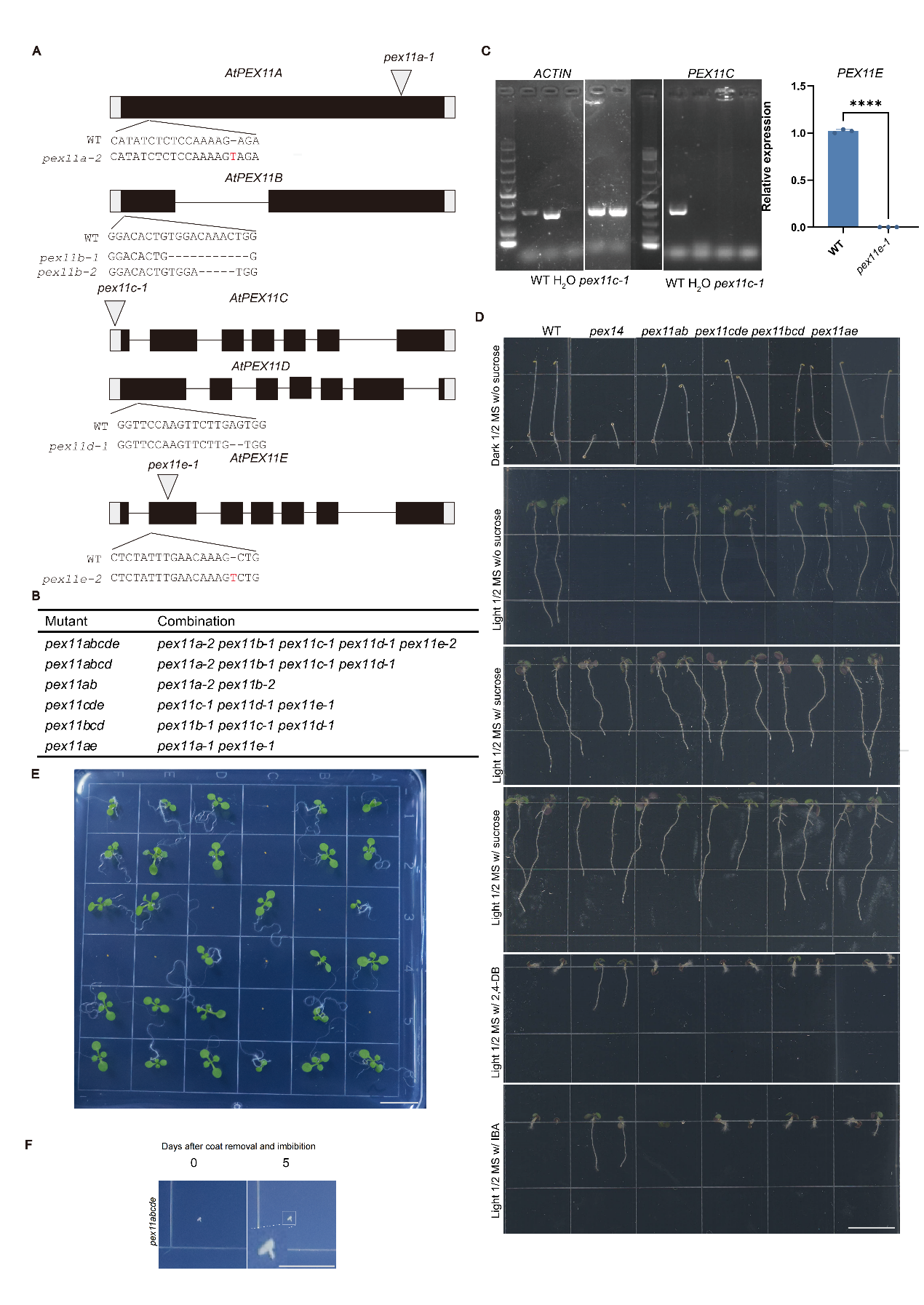
**

**Fig. S2. Mutants used on this study.**

**(A)** Schematics of the *PEX11* genes. Gray triangles indicate the T-DNA insertion sites. Thin black lines indicate the CRISPR-mediated mutation sites in the Col-0 background; nucleotides inserted in the mutants are in red. Exons, UTRs, and introns are indicated by black boxes, gray boxes, and thick black lines, respectively. **(B)** The combination of different *pex11* mutant. **(C)** Knockout analysis of *pex11* mutant. Left panel, Reverse Transcription-PCR (RT-PCR) of *pex11c-1* mutant. Right panel, Quantitative Real-time PCR (qPCR) of *pex11e-1* mutant. All values represent mean ± SD, n = 3, and significant differences were determined by two-tailed Student’s t tests (****p < 0.0001). **(D)** The phenotype images of the WT and mutants under

sucrose treatment and peroxisomal β-oxidation assays, alongside with the control. **(E)** A representative plate showing the lack of germination for approximately one quarter of the seeds from a plant heterozygous for the *pex11e* allele in a homozygous *pex11abcd* quadruple mutant background. Bar = 1 cm. (F) The phenotype of quintuple mutant seedling following seed coat removal, grown on 1 % sucrose. The inset shows a 2X enlargement of the image. Bar = 1 cm.

**Supplementary Tables**

**Table S1. The gRNA for CRISPR/Cas9 mutants.**

| Gene name | gRNA |
| --- | --- |
| *PEX11A* | CATATCTCTCCAAAAGAGA |
| *PEX11B* | GGACACTGTGGACAAACTGG |
| *PEX11D* | GGTTCCAAGTTCTTGAGTGG |
| *PEX11E* | CTCTATTTGAACAAAGCTG |

**Table S2. The primers used on this study.**

| Gene name | Forward primer | Reverse primer | Purpose |
| --- | --- | --- | --- |
| *PEX11C* | CTAGCCAAGCTGGTGTTCTTG | *TGTCGGAGATGGAAGTAATGG* | T-DNA insertion genotyping |
| *PEX11E* | ATGATCGATTGGCTGTGCTAG | *TTGTTTCTATGTCGCTCCCTG* | T-DNA insertion genotyping |
| *PEX11E* | GCCTAAAGGGACTCCACTTCC | AGCTCACCAATCTCGACTGC | RT-qPCR |
| *UBC9* | GACCCGTTGCGGAAGACAT | AGGACTCCACTGCTCCTTCA | RT-qPCR |
